# Editorial: Pharmacological actions of drugs in the brain: exploring the intricacies and potential therapeutic applications

**DOI:** 10.3389/fphar.2024.1486719

**Published:** 2024-09-11

**Authors:** Christina Dalla, Nouria Lakhdar-Ghazal, Tanya Calvey, Giuseppe Di Giovanni

**Affiliations:** ^1^ Second Department of Obstetrics - Gynecology, Aretaieio Hospital, School of Medicine, National and Kapodistrian University of Athens, Athens, Greece; ^2^ Mohammed V University Rabat, Rabat, Morocco; ^3^ Department of Human Biology, Faculty of Health Sciences, University of Cape Town, Cape Town, South Africa; ^4^ Neuroscience Institute, University of Cape Town, Cape Town, South Africa; ^5^ Department of Medical and Surgical Sciences, University of Magna Graecia, Catanzaro, Italy

**Keywords:** neuropharmacology, brain-drug interactions, therapeutic applications, neurological disorders, signal pathways

The brain’s intricate and dynamic nature has made it a focal point of pharmacological research, with the goal of understanding how various drugs interact with its complex systems (Agid et al., 2007). This Research Topic, “Pharmacological Actions of Drugs in the Brain: Exploring the Intricacies and Potential Therapeutic Applications,” brings together a diverse Research Topic of 13 articles, each shedding light on different aspects of this field. Notably, this Research Topic is part of a Research Topic dedicated to the Mediterranean Neuroscience Society Conference 2023, held in Tunis, Tunisia (MNS, 2023). These studies explore the mechanisms by which drugs modulate brain activity and behavior, offering insights into potential therapeutic avenues for a range of neurological and psychiatric conditions.

The first study in this Research Topic by Maric et al. explores the neural substrate for ghrelin’s effects on ingestive, motivated, and anxiety-like behaviors, tracing its journey from the stomach to the locus coeruleus. This study highlights the complexity of gut-brain interactions and the potential of targeting such pathways for therapeutic interventions in conditions such as anxiety and eating disorders, with a degree of sex divergence.

In another significant contribution, Karimi et al. investigate the effects of umbelliprenin, a natural coumarin product, on mitigating autistic-like behaviors in a mouse model of maternal separation stress. The study’s findings, which involve the modulation of MECP2 expression and oxidative stress, underscore the therapeutic potential of targeting epigenetic and oxidative pathways in autism spectrum disorders.

Traditional Chinese medicine (TCM) has long been a source of pharmacological exploration, and the review by Lv et al. investigates into the pharmacological mechanisms of antidepressant active ingredients in TCM. By focusing on adult hippocampal neurogenesis, this review provides a comprehensive overview of how ancient medicinal practices could be integrated within modern neuroscience, in order to address depression.

Anesthesia, a cornerstone of modern medicine, also features prominently in this Research Topic. Huang et al.’s brief research report reveals how propofol-induced anesthesia directly inhibits glutamatergic neurons in the lateral hypothalamus, offering new insights into the neural mechanisms underlying anesthetic states and their potential long-term effects on brain function.

The role of oxidative stress in brain pathology is further explored by Zhu et al., who demonstrate the protective effects of Edaravone dexborneol in experimental subarachnoid hemorrhage. This study contributes to the growing body of evidence supporting the Keap1/Nrf2 signaling pathway as a target for neuroprotective strategies.

Depression, a leading cause of disability worldwide, is examined in the context of gut-brain interactions by Li et al., who show how quercetin reshapes gut microbiota and modulates brain metabolism to regulate depression-like behaviors. This research underscores the importance of considering the gut-brain axis in developing novel antidepressant therapies.

The neurobiological effects of drugs also extend to substance abuse, as demonstrated by Govender et al., who investigate the impact of ibogaine on myelination markers following morphine administration. Their findings suggest a potential role for ibogaine in addressing the neurobiological underpinnings of addiction and its associated neural changes.

Hudetz’s study on macrostimulation and anesthetic state-dependent, effective connectivity of cortical neurons offers a novel approach to understanding how anesthesia alters neural circuits, with implications for improving anesthesia techniques and patient outcomes.

The therapeutic potential of natural compounds is further explored in the mini review by Wang et al., which examines the antidepressant activity of mushroom and fungus extracts in rodent models. This scoping review highlights the promise of these natural products in developing new treatments for depression.

Alotaibi et al. investigate the neurotoxic effects of pyrethroids and the protective role of chitosan-encapsulated curcumin nanoparticles, using a combination of morphometric, immunofluorescence, and *in silico* approaches. Their study provides valuable insights into the potential of nanoparticle-based therapies in neurodegenerative diseases.

The complex pharmacological effects of stimulant medications in adults with ADHD are explored by Thunberg et al., who provide a nuanced analysis of the categorical and dimensional aspects of these effects. Their work contributes to a better understanding of how stimulants affect brain function in both affected and healthy populations.

Finally, Yang et al. present a systematic review on the roles and mechanisms of natural herbal extracts in treating cerebral ischemia, offering a comprehensive overview of how these extracts can be harnessed to protect the brain from ischemic damage.

Closing this Research Topic, Li et al. explore the neuroprotective effects of lutein, demonstrating its ability to inhibit glutamate-induced apoptosis in HT22 cells via the Nrf2/HO-1 signaling pathway. This study reinforces the potential of targeting oxidative stress pathways in developing therapies for neurodegenerative diseases.

Together, these 13 articles not only deepen our understanding of the pharmacological actions of drugs in the brain, but also highlight the potential for developing new therapeutic strategies to address a range of neurological and psychiatric disorders. The insights gained from these studies are poised to inform future research and clinical practice, paving the way for more effective treatments and better patient outcomes.

We hope that more discussion and insights will be presented at the next Mediterranean Neuroscience Society Conference 2025, held in Crete, Greece. For more details, please visit https://www.medneuroscisociety.org/.
